# Nutrological therapy in oncology: from prevention to nutritional support during treatment

**DOI:** 10.1590/1806-9282.2024S123

**Published:** 2024-06-07

**Authors:** Alexandre Nogueira Matos, Simone Chaves de Miranda Silvestre, Sandra Lúcia Fernandes, Aritana Alves Pereira, Rodrigo Fernandes Weyll Pimentel, Marina Carvalho-Rassbach, Ligia Carvalho de Albuquerque, Nelson Iucif

**Affiliations:** 1 Brazilian Association of Nutrology – Catanduva (SP), Brazil.; 2 Hospital Felicio Rocho – Belo Horizonte (MG), Brazil.

## INTRODUCTION

Protein-calorie malnutrition is highly prevalent in oncology patients, particularly in the advanced stages of the disease, with an estimated 20–30% of patient deaths attributed to this nutritional state^1^. Loss of lean body mass, muscle strength, and performance, along with increased morbidity and mortality, reduced quality of life, and lower tolerance to treatment toxicity leading to more frequent treatment interruptions, are characteristic features of malnutrition^1,2^. Its causes may stem from the cancer itself and/or its treatment and encompass factors such as reduced food intake, increased energy expenditure, metabolic disturbances related to the tumor, and, in some cases, malabsorption. Hyporexia can occur in 40–80% of cases and is multifactorial, potentially arising from issues like nausea, dysgeusia, dysphagia, pain, and depression, among others.

Given the high prevalence of malnutrition, all patients should be assessed to detect nutritional risk and be reassessed throughout their treatment journey^3^. Nutritional care ranges from prevention to nutritional therapy for cachexia and the follow-up of survivors. Furthermore, it is of paramount importance in newly diagnosed cases. Unfortunately, it is estimated that only 30–70% of oncology patients at risk of malnutrition are evaluated, and only about half of them receive appropriate intervention^4^.

## SCREENING AND DIAGNOSIS

Every patient should be evaluated to determine their nutritional risk and status at the time of oncology diagnosis and periodically reassessed during each stage of treatment^3^. To screen for nutritional risk, a validated tool called Nutritional Risk Screening–2002 (NRS-2002), developed by Kondrup et al.^3^ and certified by the European Society for Parenteral and Enteral Nutrition (ESPEN), is utilized.

The patient's nutritional status correlates with the severity of the disease and its prognosis. Patients at nutritional risk benefit more from nutritional therapy^3^. Nutritional screening and diagnostic tools correlate the cause and consequence effects of malnutrition. The causes include reduced food intake, poor absorption, or increased energy expenditure, while the consequences encompass weight loss, low body mass index (BMI), and loss of body compartments.

In NRS-2002, all cause and consequence variables are scored according to their severity. The sum of these scores, if greater than 3, indicates nutritional risk. Elderly patients receive an additional point in NRS-2002 due to their inherent age-related risk^3^. The initial stage involves asking four questions (Stage 1), and if the answer is “yes” to any of them, one should proceed to the subsequent stage (Stage 2).

## NUTRITIONAL DIAGNOSES IN ONCOLOGY

Possible nutritional diagnoses in oncology include adequate nutritional status, sarcopenia, malnutrition, and cachexia^5^. Sarcopenia is a skeletal muscle disorder that correlates with the risk of falls, fractures, physical disability, and mortality^6^. It is also described as a geriatric syndrome, and its diagnosis relies on the assessment of muscle strength, mass, and performance^7^. Cachexia is associated with a catabolic state, loss of lean mass, and often a decrease in fat mass. It affects the majority of advanced cancer patients, reducing treatment tolerance, efficacy, quality of life, and survival. It can also occur in other conditions besides cancer, such as cardiac cachexia in heart failure or pulmonary cachexia in patients with chronic obstructive pulmonary disease^5^.

## DIAGNOSIS OF CACHEXIA^5^

Malnutrition can occur in any medical condition, with unintentional weight loss being one of its key criteria (Table 1).

**Table 1:** Cachexia diagnosis criteria.

Pre-cachexia	Cachexia	Refractory cachexia
All of the criteria	One or more criteria	One criterion
Weight loss	Weight loss >5% over past 6 months	Limited self-care
Clinical Symptoms (i.e., anorexia)	Weight loss >2% and BMI <20	Complete disability on WHO performance status and life expectancy less than 3 months
Metabolic symptoms (i.e., glucose intolerance)	Weight loss >2% and sarcopenia as detected by MUMA or ASMI by DXA, or LSMI by CT, or FFMI by BIA*	

* MUMA: mid upper-arm muscle area – reference values: men <32 cm^2^, women <18 cm^2^; ASMI: appendicular skeletal muscle index – reference values: men <7·26 kg/m^2^; women <5·45 kg/m^2^; DXA: dual energy x-ray absorptiometry; LSMI: lumbar skeletal muscle index – reference values: men <55 cm^2^/m^2^; women <39 cm^2^/m^2^; CT: computed tomography; FFMI: fat-free mass index without bone – reference values: men <14·6 kg/m^2^; women <11·4 kg/m^2^; BIA: bioelectrical impedance; WHO: World Health Organization.

For the diagnosis of malnutrition, the Global Leadership Initiative on Malnutrition (GLIM; Table 2)^8^ has proven to be an excellent tool with good specificity and sensitivity. It is based on phenotypic and etiological criteria encompassing various mechanisms and causes of malnutrition. In GLIM, different instruments for assessing body composition can be used to evaluate muscle mass, such as dual-energy X-ray absorptiometry (DEXA), bioimpedance, or computed tomography.

**Table 2:** Global leadership initiative on malnutrition^8^.

Phenotypic criteria			Etiologic criteria	
Weight loss (%) in 6 months	Low body index	Reduced muscle mass	Reduced food intake or assimilation	Inflammation
>5%: moderate malnutrition or >10%: severe malnutrition	<20 kg/m^2^ if <70 yo; <22 kg/m^2^ if >70 yo; Asiatic population: <18.5 kg/m^2^ if <70 yo; <20 kg/m^2^ if > 70 yo;	Diagnosed by validated body composition measuring techniques	≤50% of RER for 1 week or any reduction for >2 weeks or any chronic GI condition that adversely impacts food assimilation or absorption	Acute disease/injury or chronic disease-related

RER: resting energy requirements; GI: gastrointestinal.

## LABORATORY TESTS

In addition to the previously mentioned diagnostic tools, some biochemical and imaging tests can be added to support the nutritional diagnosis of oncology patients, using them as prognostic indicators. The preoperative serum albumin level is inversely related to a higher incidence of fistulas and postoperative infectious complications^9^. Some studies use the perioperative total serum protein value as part of the risk calculation for anastomotic fistulas^10^. The Glasgow Prognostic Score is another example of a prognostic calculation where the serum albumin level associated with the C-reactive protein value reflects the nutritional status and degree of inflammation in patients with various types of cancer^11^.

## NUTRITIONAL MANAGEMENT AT THE TIME OF DIAGNOSIS

Nutritional management is essential for preventing losses, providing support, or facilitating recovery. It is a non-pharmacological and essential adjuvant approach for chemotherapy (CT), radiotherapy (RT), immunotherapy, and/or surgery. Approximately 80% of solid tumor patients will undergo some form of surgical treatment. According to ESPEN Guidelines^1^, oncology patients diagnosed with malnutrition should be nutritionally prepared for surgery. The optimization of daily calorie intake should be calculated at around 25–30 kcal/kg and above 1.0 g of protein/kg, reaching 1.5 g/kg^1^.

Nutritional therapy to achieve this goal includes hyperprotein and high-calorie oral supplements when the patient can consume more than 60% of their caloric needs orally. Nasoenteral feeding should be indicated when intake is less than 60% of requirements. In cases where gastrointestinal (GI) function is partially compromised or intestinal failure occurs, supplemental or total parenteral nutrition, respectively, is recommended^1^. For patients with a surgical perspective, a minimum prehabilitation of 7–14 days before surgery should be performed, and it may be delayed for this purpose^1,12^. This approach has also been studied for other oncological treatments, including CT and RT, with benefits in terms of morbidity, mortality, and hospitalization time^12^. Currently, studies have suggested that the use of probiotics for 3–8 days in the preoperative period of colorectal cancer patients could reduce postoperative infectious complications and improve intestinal permeability with a reduction in bacterial translocation^13^. For malnourished patients with upper GI neoplasms, there is moderate evidence for the use of supplements enriched with omega-3, arginine, and nucleotides^1,12^.

The systematic multimodal approach of Enhanced Recovery After Surgery (ERAS^14^) and the ACERTO project^12^ place nutritional therapy in a prominent position as a measure that can modify surgical outcomes.

Prehabilitation is now recommended as a preparation for CT, RT, and immunotherapy as well. Special attention should be given to patients with head and neck cancers undergoing RT. These patients often experience a drastic deterioration in their nutritional and hydration status, leading to treatment interruptions and frequent hospitalizations. Prehabilitation protocols include clinical, psychological, nutritional, and physiotherapeutic preparation to ensure that the patient is in the best possible clinical condition for the proposed therapy, whether surgical, CT, or RT^12^. Managing depression is of paramount importance, as it can impact food intake, the ability to engage in physical therapy, and the patient's prognosis. Physiotherapeutic intervention aims to stimulate protein synthesis and prepare the patient both physically and respiratory-wise for the postoperative period. Prehabilitation should begin at the time of oncology diagnosis and last between 2 and 6 weeks when surgery allows for this waiting period^15^. Attention should be paid to patients with a high body mass index and neoplasia, as there is a high incidence of sarcopenic obesity, which can affect prognosis and treatment outcomes^16^.

## NUTRITIONAL MANAGEMENT DURING TREATMENT

The tumor has various effects on the GI tract. Up to 50% of patients experience taste changes at the time of diagnosis, directly affecting food intake^1^. In addition to this factor, CT and RT cause side effects such as oral mucositis, nausea, vomiting, diarrhea, and sarcopenic dysphagia. About 35% of patients experience GI symptoms during CT and RT, and more than 60% experience late-onset symptoms^17^. GI symptoms should be prevented and treated, and menu adjustments and consistency modifications can help improve oral diet acceptance^18^. Another important approach is to assess hedonic changes of taste and provide diets adapted to that change focusing at increasing ingestion.^19^ Recurrent fasting for tests and procedures can also compromise intake. Malnutrition is a key determinant of worse outcomes in cancer treatments^18^. One significant challenge in managing these patients is how to prevent or delay the onset of refractory cachexia. Due to the inflammatory process triggered by the tumor and its resulting metabolic changes, medication interventions are necessary to improve anorexia. Some options include the use of omega-3, preferably eicosapentaenoic acid (EPA), at a dose of 600 mg to 2.2 g/day. However, there are varying doses in the literature, ranging from 1 g to 3 g/day of a combination of EPA and docosahexaenoic acid (DHA) in a ratio of 1.5:2.0. There is evidence for the use of mirtazapine at 15–45 mg/day or olanzapine at 5 mg/day in the treatment of anorexia. Although some evidence suggests the use of megestrol acetate and corticosteroids, these are not considered first-line options due to their side effects: 1 in 4 patients experiences increased appetite, 1 in 12 experiences weight gain, but 1 in 6 develops thromboembolic events, and 1 in 23 dies as a result of the medication^20^.

## NUTRITIONAL MANAGEMENT IN PREVENTION

Among the various factors related to cancer are chronic inflammation, oxidative stress, cell cycle changes, and the activation of pro-oncogenes. The quality of nutrition significantly influences the risk of developing cancer. Preventive measures based on lifestyle changes, including physical activity and a healthy diet, can impact around 3–4 million new cases worldwide^21^. The Mediterranean diet (MD) is the most studied in oncological prevention.

### The Mediterranean diet

The potential for reducing the risk of cancer through the MD has been extensively studied due to its profile of anti-inflammatory foods, including antioxidants, polyphenols, and omega-3 fatty acids (FAs). Its antioxidants and polyphenols are associated with an antineoplastic effect^22^.

This diet is primarily based on plant-based foods such as fresh fruits, unrefined grains, nuts, whole grains, seafood, olive oil, and low-fat dairy products such as cow's milk and low-fat cheeses. The consumption of wine is permitted, while the consumption of red meat and sugar should be occasional^22^.

The MD is rich in fruits, vegetables, and legumes, which are abundant sources of antioxidants such as vitamin C and E, as well as phytochemicals like flavonoids and carotenoids^23^. These components neutralize the harmful effects of free radicals in the body, which can cause DNA damage and increase the risk of cancer^24^.

The MD includes healthy fats from sources like olive oil, nuts, and fish. This provides an adequate intake of polyunsaturated omega-3 fatty acids (PUFAs) and monounsaturated omega-9 FAs. This profile of FAs can reduce inflammation, a factor correlated with oncological diseases.

The variety of grains, legumes, and pulses in the MD ensures an adequate intake of fiber. Fiber aids in digestion, maintains a healthy microbiota, and has the potential to reduce the risk of colorectal cancer.

The consumption of red meat in the MD is reduced, with a preference for lean meats such as poultry and fish. High consumption of red and processed meat is associated with the risk of certain cancers, including colorectal cancer^25^.

Moderate wine consumption in the MD may protect against certain neoplasms, although it is known that there is no safe minimum dose of ethanol intake^26^. The Mediterranean lifestyle also includes regular physical activities and group activities, which contribute to well-being and can reduce the risk of cancer.

Weight control can be achieved even with normocaloric diets, as there is better appetite control through diets with higher nutritional density. Maintaining a healthy weight is essential to reduce the risk of various types of cancer, including breast, colorectal, and endometrial cancers^27^.

Some nutrients in the MD can act protectively through epigenetics, inhibiting tumor development. The MD can protect against metabolic and oxidative DNA damage^28^.

In addition to the Mediterranean lifestyle, smoking should be ceased, and exposure to environmental carcinogens should be reduced.

### Diets rich in vegetables

The diets that are rich in vegetables include paleolithic (PD) and vegetarian (VD). The PD consists of consuming foods that our ancestors would have used during the Paleolithic era. It includes the consumption of lean meats, fish, fruits, vegetables, nuts, and seeds while avoiding processed foods, grains, dairy, and legumes. There are theories that the PD may bring health benefits and prevent cancer^29^, but caution should be exercised in drawing conclusions for this purpose.

When well balanced, the VD can also have benefits in reducing the risk of cancer. This is partly due to the benefits of plant-based foods with their antioxidant components, but also due to the restriction of animal product consumption^30^. Additionally, in the VD, there is a reduction in exposure to hormones and antibiotics, which are common in dairy and meat products^31^. The consumption of fruits and vegetables, as well as the restriction of meat consumption, show benefits, as previously mentioned in the MD^31^. However, attention should be paid to the adequate intake of vitamin B12, iron, calcium, and protein.

Both diets are rich in vitamins, minerals, and antioxidants, which can prevent DNA damage and reduce the risk of cancer^32^. Nevertheless, well-designed studies that can recommend these diets for cancer prevention are still needed.

### Ketogenic diet

Diets based on fasting have been extensively studied, including the ketogenic diet (KD), fasting itself, and the fasting mimicking diet. The KD is a high-fat, low-carbohydrate diet that can assist in cancer prevention and treatment and may have anticancer effects.

The theoretical premise is that neoplastic cells use glucose as an energy substrate through the Warburg effect, which involves aerobic fermentation (or aerobic glycolysis). By depriving the oncological cell of carbohydrates, its development and survival can be compromised. The KD could reduce chronic inflammation, which is correlated with oncogenesis, tumor growth, invasion, and insulin resistance^33^.

It is important to note that despite the benefits demonstrated in vitro, we currently lack sufficient clinical studies, and there is no scientific evidence supporting the use of this diet during cancer treatment^34^. Additionally, the KD, like other restrictive diets, is difficult to sustain in the long term. It can also lead to nutritional deficiencies, constipation, and cardiovascular risk if not closely monitored by a nutrologist or healthcare professional^35^.

### Foods to be avoided

For the prevention of cancer, reducing the consumption of saturated and trans fats is recommended to lower the risk of breast and colorectal cancer. This includes avoiding processed meats such as bacon and sausages, which are associated with colorectal cancer^36^.

According to the World Health Organization and the International Agency for Research on Cancer, dietary patterns based on the regular consumption of fruits, vegetables, and foods rich in selenium, folic acid, vitamins (B12 or D), and antioxidants (such as carotenoids and lycopene) play a protective role in cancer prevention^37^ and should be prioritized in a healthy diet. Omega-3, abundant in fish, especially sardines and mackerel, can help slow down the development of cancer^38^.

A high intake of fiber-rich products (e.g., whole grains) and moderate consumption of milk and dairy products can reduce the incidence of various types of cancer, including colorectal, lung, stomach, breast, esophageal, and oral cancer^39,40^.

## CONCLUSION

Cancer is one of the leading causes of mortality and disability in today's world. Antineoplastic treatments are advancing gradually; however, from a nutritional standpoint, early diagnosis of malnutrition and the introduction of nutritional therapy as soon as necessary remain crucial. Prevention focuses on a healthy lifestyle with a diet rich in vegetables, unsaturated fats, and complex carbohydrates, coupled with regular physical activity.
